# Mortality Trends in Pediatric Hepatoblastoma: A Brazilian and Global Perspective

**DOI:** 10.3390/cancers17182970

**Published:** 2025-09-11

**Authors:** Raquel Francine Liermann Garcia, Camila Barbosa, José Guilherme Pickler, Francis Rosseti Pedack, Christian Evangelista Garcia, Hercilio Fronza Junior, Bruna Louise Silva, Paulo Henrique Condeixa de França, Bárbara Sarni Sanches, Marcelo Gerardin Poirot Land, Rafael Roesler, Karina Munhoz de Paula Alves Coelho

**Affiliations:** 1 Department of Scientific Development and Innovation (DECIPE), Center for Anatomo-Pathological Diagnosis (CEDAP), Joinville 89201-330, SC, Brazil; raquel.garcia@univille.br (R.F.L.G.); jose.pickler@univille.br (J.G.P.); bl.silva@edu.udesc.br (B.L.S.); 2 Graduate Program in Health and Environmental Sciences, University of the Joinville Region (UNIVILLE), Joinville 89219-710, SC, Brazil; 3 São José City Hospital, Joinville 89202-030, SC, Brazil; 4 Department of Mechanical Engineering, Center for Technological Sciences, Santa Catarina State University (UDESC), Joinville 89219-710, SC, Brazil; 5 National Science and Technology Institute for Children’s Cancer Biology and Pediatric Oncology-INCT BioOncoPed, Porto Alegre 90035-003, RS, Brazil; barbarasarni@ufrj.br (B.S.S.); rafaelroesler@hcpa.edu.br (R.R.); 6 Department of Clinical Medicine, Faculty of Medicine, Federal University of Rio de Janeiro, Rio de Janeiro 21941-902, RJ, Brazil; 7 Martagão Gesteira Childcare and Pediatrics Institute, Federal University of Rio de Janeiro (IPPMG-UFRJ), Rio de Janeiro 21941-912, RJ, Brazil; 8 Department of Pharmacology, Institute for Basic Health Sciences, Federal University of Rio Grande do Sul, Porto Alegre 90035-003, RS, Brazil; 9 Cancer and Neurobiology Laboratory, Experimental Research Center, Clinical Hospital (CPE-HCPA), Federal University of Rio Grande do Sul, Porto Alegre 90035-003, RS, Brazil; 10 Graduate Program in Medical Sciences, Faculty of Medicine, Federal University of Rio Grande do Sul, Porto Alegre 90035-003, RS, Brazil

**Keywords:** hepatoblastoma, liver tumor, pediatric cancer, cancer mortality, pediatric oncology

## Abstract

*Background:* Hepatoblastoma is a rare malignant liver tumor that accounts for 1–2% of pediatric cancers. Despite its low incidence, it is a significant cause of cancer-related mortality in early childhood. *Methods:* This ecological study analyzed hepatoblastoma mortality in Brazilian children and adolescents (2008–2023) using data from the Mortality Information System (SIM/DATASUS). Mortality rates were calculated using official population estimates. Temporal trends were assessed using Prais-Winsten regression. Age and sex differences were analyzed using the chi-square test. Global mortality estimates (2008–2021) were obtained from the Global Burden of Disease Study for descriptive comparison. *Results:* A total of 267 deaths were recorded, most (66.7%) in children aged 0–4 years. Males accounted for 61.4% of cases. Although no significant mortality trends were observed for younger age groups, a significant annual decline was found among adolescents aged 15–19 years (Annual Percent Change (APC) = −38.4%, *p* = 0.016). Regional disparities were evident, with the Southeast presenting the highest number of deaths. Globally, the estimated number of deaths and age-specific mortality rate (ASMR) decreased over time, particularly among children under five. *Conclusions:* Hepatoblastoma remains a significant cause of mortality in early childhood. While Brazilian mortality rates remained stable in younger groups, mortality in adolescents showed a marked reduction. Global estimates suggest a progressive reduction in mortality.

## Simple Summary

Hepatoblastoma is a rare type of liver cancer that mostly affects young children. Even though it does not occur often, it is one of the main causes of cancer-related deaths in early childhood. This study looked at deaths caused by hepatoblastoma in children and teenagers in Brazil over the past 16 years. The goal was to understand how often these deaths occur, if they have increased or decreased over time, and whether there are differences between age groups, sexes, or regions of the country. The study found that most deaths happened in children under five years old and were more frequent in boys. Although death rates remained stable in young children, there was a clear decrease among teenagers. The results also showed regional differences, with some parts of the country being more affected. These findings help improve our understanding of this cancer and may guide future public health strategies to reduce its impact.

## 1. Introduction

Hepatoblastoma is a rare embryonal neoplasm likely originating from hepatocyte precursor cells, often resembling stages of liver tissue development, which accounts for only 1% to 2% of all pediatric tumors [1]. However, it remains the most common hepatic neoplasm in childhood, representing 37% of primary liver tumors in this population. Its incidence reaches 1.2–1.5 cases per million, peaking in children under three years of age and becoming less frequent after five years old, with a higher prevalence in males [2]. 

In recent decades, there has been a remarkable global increase in the number of hepatoblastoma cases, possibly associated with rising rates of prematurity and low birth weight, conditions well-recognized as risk factors for this neoplasm [3]. Other predisposing conditions include genetic syndromes, such as Beckwith-Wiedemann syndrome and familial adenomatous polyposis, parental smoking, and other maternal pathologies [4].

Hepatoblastoma is characterized by a low incidence of genetic variants and is considered a disease primarily driven by the activation of the WNT (wingless-related integration site) signaling pathway. Most cases exhibit recurrent mutations in catenin beta 1 (CTNNB1) gene, alongside some mutations in nuclear factor erythroid 2-related factor 2 (NFE2L2). Although variants of NFE2L2 are relatively rare, they are linked to increased resistance to chemotherapy and lower survival rates. Mutations in telomerase reverse transcriptase (TERT) and tumor protein p53 (TP53) genes are rare; however, when present, they contribute to the aggressive nature of the disease [5,6,7]. 

The typical clinical presentation is a large abdominal mass, often accompanied by obstructive symptoms of the hepatobiliary tract [3]. Laboratory analysis usually reveals elevated alpha-fetoprotein (AFP) levels associated with thrombocytosis, often without changes in liver enzymes [8]. Diagnosis is based on clinical presentation and imaging examination, complemented by serum AFP measurement, which is the most important marker for diagnosis, therapeutic response assessment, and follow-up of hepatoblastoma [9]. Pretreatment staging is performed using the PRETEXT system, which considers criteria as the presence of metastatic disease, multifocality, and vascular or extrahepatic invasion [10]. The overall prognosis shows a 5-year survival rate of 70%, with worse outcomes associated with high PRETEXT scores and elevated AFP levels [11].

Due to its rarity, hepatoblastoma remains poorly characterized in population-based studies of pediatric cancer. This study aims to evaluate national mortality patterns related to hepatoblastoma in Brazilian children and adolescents over a 16-year period, exploring temporal trends, age-specific and regional distributions, and contextualizing the findings within global estimates.

## 2. Materials and Methods

This ecological study was based on a retrospective, descriptive, and exploratory analysis of pediatric hepatoblastoma mortality data in Brazil from 2008 to 2023. It also included global mortality estimates from 2008 to 2021. The study also comprised three complementary analytical components: a descriptive analysis of mortality data and associated demographic profile, a statistical evaluation of temporal trends and age group differences, and a comparative analysis based on global estimates. For time-series analysis, we applied Prais-Winsten regression using the prais package in R version 1.1.4. [12,13]. For graphical representation of time-series data, we used the ggplot2 package version 3.5.2. [14] in the same environment.

### 2.1. Data Sources and Processing of Brazilian Data

Mortality data is anonymous and was collected from the national registry of the Mortality Information System (SIM), provided by the Department of Informatics of the Unified Health System (DATASUS), of Brazil and its 27 federative units. Brazilian federative units were grouped into five macro-regions—North, Northeast, Southeast, South, and Mid-West, according with Brazilian Institute of Geography and Statistics (IBGE). Consolidated data from 2008 to 2023, including preliminary data from 2023, were collected on 24 September 2024.

The variables extracted from SIM reflect the information on the Death Certificate (DC), including year and date of death, date of birth, age at death, gender, race, federal unit, underlying cause, and items A (Underlying cause of death), B (Intermediate causes), C (Contributory causes), and D (Additional conditions or underlying factors) from the DC.

Demographic information and population indices were obtained through the Application Programming Interface (API) of the IBGE, integrating census data and official population estimates. The average mortality rate was calculated for the total pediatric population (0 to 19 years) and for the age groups of 0 to 4, 5 to 9, 10 to 14, and 15 to 19 years using corresponding data from IBGE.

### 2.2. Descriptive Statistical Analysis of Brazilian Data

The variables analyzed included year of death, age group, sex, race/skin color, and geographic region of residence. Absolute numbers, proportions, and age-specific mortality rates (ASMR) per 1,000,000 population were calculated based on population estimates from the Brazilian Institute of Geography and Statistics (IBGE).

Temporal and demographic patterns were assessed to identify potential disparities and trends in the national context. To support the interpretation of findings, results were visualized through graphical representations, allowing for a comprehensive overview of variations over time and across demographic categories.

### 2.3. Exploratory Statistical Analysis of Brazilian Data

Time series analysis was conducted for Brazil using the Prais-Winsten (PW) estimation method. The general structure of a Prais-Winsten regression can be defined as Equation (1).

Yt = α + β × Xt + ϵt (1)

where Yt is the dependent variable, α is the intercept, Xt is the independent variable, and ϵt is the error term. This approach is used to estimate the correlation between errors at time t and t−1, and these errors should follow a first-order autoregressive (AR (1)) process. 

Other models were tested: simple linear, log-linear, quadratic, exponential and generalized additive models. However, the log-linear PW regression model provided a better fit (indicated by a higher R^2^), allowing for the interpretation of the percentage change in the mortality rate and satisfactorily correcting for data autocorrelation. Thus, this was the model of choice. In age groups which did not show any significant temporal autocorrelation, we did a simple log-linear regression.

For the time trend analysis, the Annual Percent Change (APC) of the ASMR was calculated using the approach proposed by Antunes [15], which is defined as Equation (2).

APC = [−1 + 10^b1^] × 100% (2)

where b1 is the slope of the independent variable (year) obtained through consistent methods for time-series calculation, as the Prais-Winsten regression [16].

To evaluate potential differences between demographic groups, comparisons were performed using the chi-square test for proportions, with a significance level set at *p* < 0.05 for all statistical tests. The comparison strategy included analyses between age strata and between sexes, aiming to identify significant disparities in the distribution of hepatoblastoma-related mortality across these demographic variables.

### 2.4. Comparative Analysis with Global Data

A complementary analysis was conducted using an ecological time-series approach to explore temporal trends in hepatoblastoma-related mortality within the global pediatric population. Mortality data for children aged 0 to 9 years were retrieved from the Global Burden of Disease (GBD) Study as provided by Global Burden of Disease Collaborative Network 2023 [17]. The analysis covered the period from 2008 to 2021, stratifying the population into two age groups: 0–4 years and 5–9 years. For each year, there were estimates of the number of deaths and corresponding ASMR (per 100,000 population). The GBD dataset employs standardized statistical models that integrate multiple data sources and enable the generation of consistent estimates, even in the presence of incomplete or heterogeneous information across countries. Thus, the values presented in this study reflect model-based estimates and should be interpreted in light of this methodological framework.

## 3. Results

The analysis followed a three-stage approach. Initially, Brazilian mortality data due to hepatoblastoma were descriptively assessed to characterize the demographic and regional profile. Subsequently, temporal trends were analyzed to identify variations in mortality over time and across age and sex groups. Finally, national findings were compared with international estimates to position Brazil within the global context of hepatoblastoma mortality.

### 3.1. Descriptive Statistical Analysis of Brazilian Data

**Figure 1:**
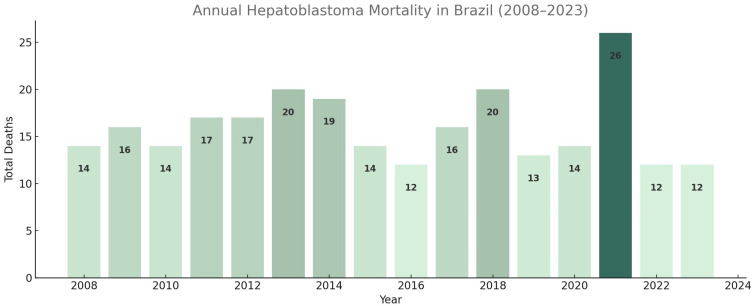
Annual number of deaths by hepatoblastoma among individuals aged 0–19 years in Brazil, 2008–2023. Data sourced from the Mortality Information System (SIM/DATASUS).

Between 2008 and 2023, a total of 267 deaths attributable to hepatoblastoma were recorded in the SIM/DATASUS mortality database, with an average of 16.7 cases per year. The highest incidence was recorded in 2022, with 26 cases (Figure 1). 

**Figure 2:**
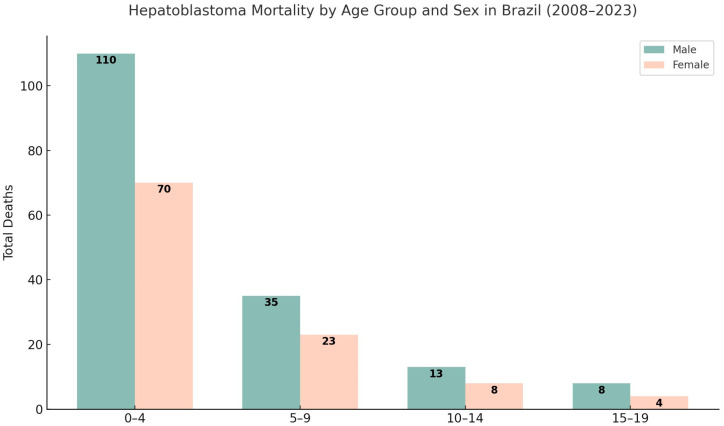
Distribution of hepatoblastoma mortality by age group and sex in Brazil, 2008–2023. This bar chart illustrates the total number of deaths due to hepatoblastoma among male and female individuals within four pediatric age groups (0–4, 5–9, 10–14, and 15–19 years) and sex. Across all age groups, mortality was consistently higher among males.

The distribution of hepatoblastoma-related deaths by age and sex reveals a marked concentration in early childhood, particularly among children aged 0–4 years, who accounted for 178 of the 267 deaths (66.7%). The number of deaths progressively decreased in older age groups: 58 in the 5–9 group (21.7%), 21 in the 10–14 group (7.9%), and 10 in the 15–19 group (3.7%). Mortality was consistently higher among males across all age groups, who accounted for 61.4 % (*n* = 164) of all deaths in the period (Figure 2).

**Figure 3:**
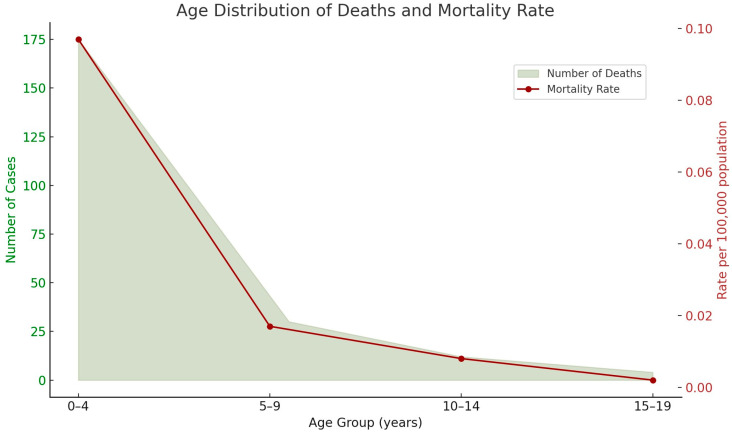
Age-specific distribution of hepatoblastoma cases and corresponding mortality rates in the Brazilian pediatric population. The majority of cases (*n* = 176) occurred in children aged 0–4 years, with a progressive decline in older age groups. The mortality rate (per 100,000 population) also followed this trend, with the highest rate observed in the youngest age group. This pattern underscores the higher incidence and burden of hepatoblastoma in early childhood.

A sharp decline in both case numbers and mortality rates with increasing age suggest that the disease primarily affects younger children. Figure 3 illustrates this trend, highlighting the predominance of cases in the 0–4-year age group and the highest mortality rate was observed in this age group.

**Figure 4:**
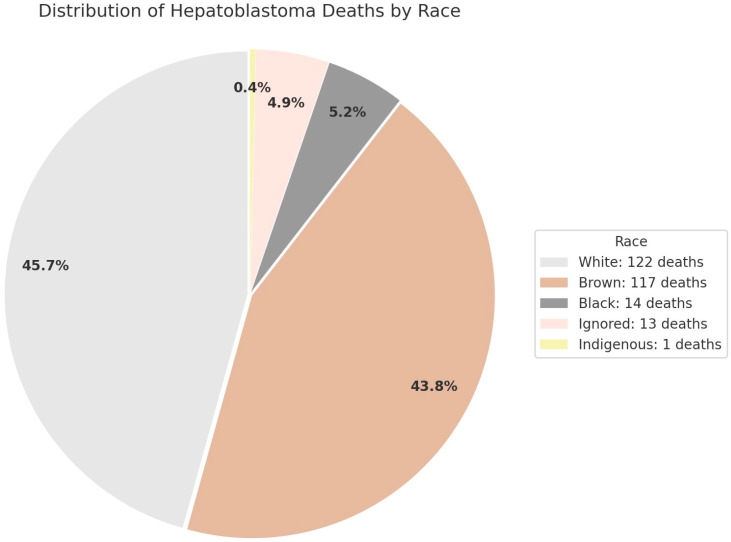
Distribution of hepatoblastoma deaths by race in Brazil, 2008–2023, in individuals aged 0–19 years, according to race as recorded in the national Mortality Information System (SIM/DATASUS). Most cases occurred among individuals identified as white, followed by brown and black. Categories “Ignored” and “Indigenous” represented smaller proportions. Labels indicate the percentage of cases in each racial group, while the legend provides absolute numbers.

Racial distribution of hepatoblastoma-related mortality reveals disparities across Brazilian population groups. Based on national mortality records, the highest proportion of deaths occurred among individuals identified as White, followed by Brown and Black populations. Smaller percentages were observed in the Indigenous and Ignored categories. These findings reflect the demographic composition of the Brazilian pediatric population and underscore the importance of considering race in analyses of disease burden and health inequities. Figure 4 summarizes this distribution in a pie chart, with percentage labels and absolute values by group.

**Figure 5:**
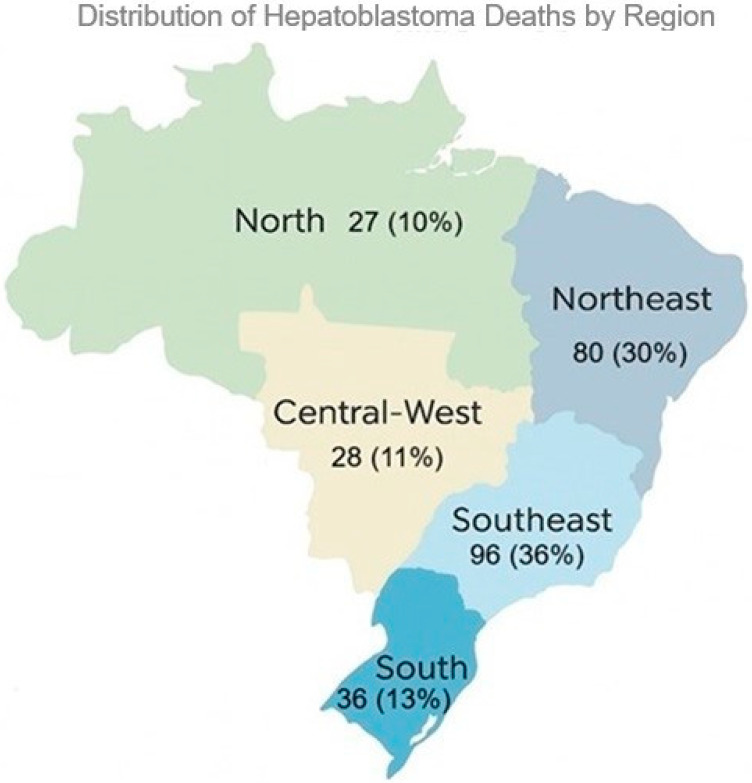
Geographic distribution of hepatoblastoma-related deaths in Brazil by macro-region. The Southeast region accounted for the highest number of deaths (96; 36%), followed by the Northeast (80; 30%), South (36; 13%), Central-West (28; 11%), and North (27; 10%).

Among the 27 Brazilian states, the highest numbers of hepatoblastoma-related deaths were recorded in São Paulo, Bahia, Rio de Janeiro, and Minas Gerais. São Paulo alone accounted for a disproportionately high number of deaths, with 53 deaths, representing approximately 19.9% of all cases. Other states such as Pernambuco, Paraná, and Ceará also contributed with notable absolute numbers. When grouped by macro-regions, the Southeast accounted for the highest number of deaths (96; 36%), followed by the Northeast (80; 30%), South (36; 13%), Central–West (28; 11%), and North (27; 10%) (Figure 5).

### 3.2. Exploratory Statistical Analysis of Brazilian Data

**Figure 6:**
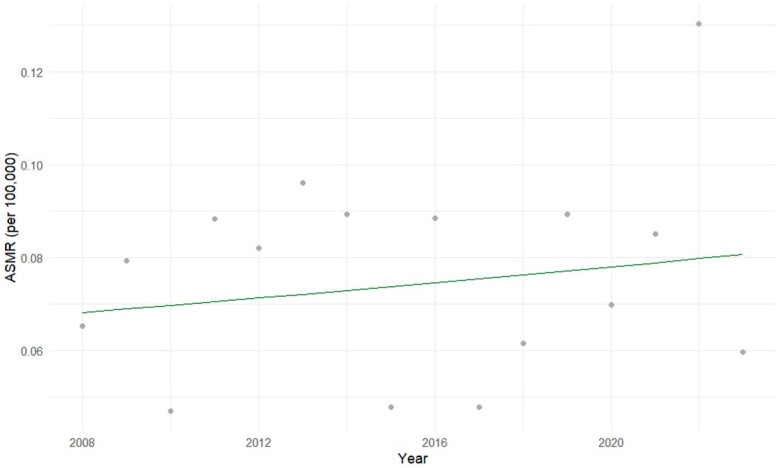
Age-specific mortality rates (ASMR) for hepatoblastoma in children aged 0 to 4 years in Brazil (2008–2023). The gray dots represent the actual ASMR for each year, and the green line represents the mortality trend estimated by the Prais-Winsten regression model. Prais-Winsten regression showed no statistically significant trend over time, despite a significant overall model (*p* = 0.017).

The Prais-Winsten regression analysis for children aged 0 to 4 years did not reveal a statistically significant temporal trend in hepatoblastoma mortality (*p* = 0.38; 95% CI), although the overall model was statistically significant (*p* = 0.017), suggesting a possible weak or non-linear relationship over time (Figure 6).

**Figure 7:**
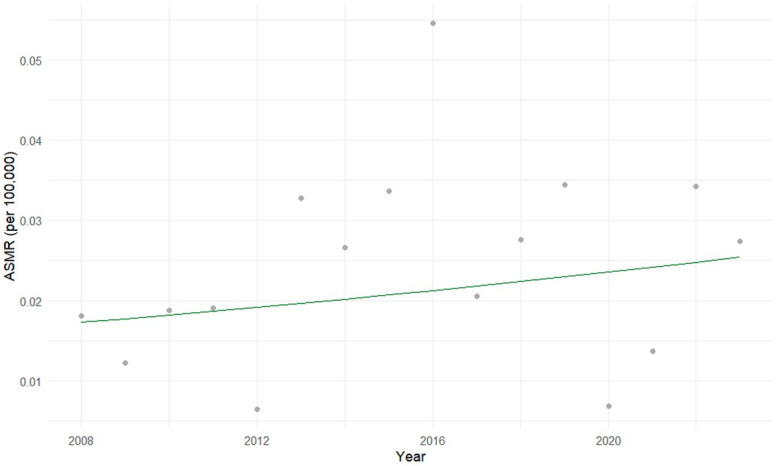
Age-specific mortality rates (ASMR) for hepatoblastoma in children aged 5 to 9 years in Brazil (2008–2023). The gray dots represent the actual ASMR for each year, and the green line represents the mortality trend estimated by the Prais-Winsten regression model. No significant temporal trend was detected using Prais-Winsten or ordinary log-linear regression models.

In the 5 to 9 year age group, the Prais-Winsten regression did not detect a statistically significant temporal trend in hepatoblastoma mortality (*p* = 0.452 95% CI), and the overall model was not significant (*p* = 0.393). Residuals showed no relevant autocorrelation (Original and Transformed Durbin-Watson statistic ≈ 2). Due to the lack of autocorrelation, an ordinary log-linear regression was also performed, which confirmed the absence of a significant temporal trend (*p* = 0.439), with a very small proportion of explained variance (adjusted R^2^ = −0.025; residual standard error = 0.5953) (Figure 7).

**Figure 8:**
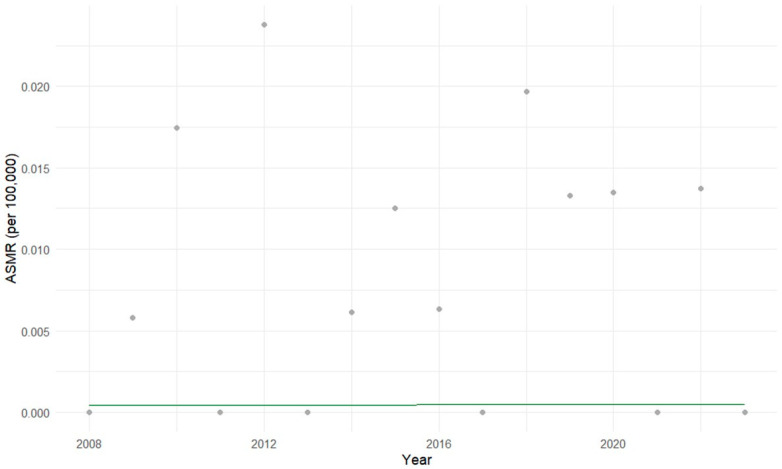
Age-specific mortality rates (ASMR) for hepatoblastoma in children aged 10 to 14 years in Brazil (2008–2023). The gray dots represent the actual ASMR for each year, and the green line represents the mortality trend estimated by the Prais-Winsten regression model. Prais-Winsten regression indicated no meaningful linear trend and minimal variation explained.

Among individuals aged 10 to 14 years, the Prais-Winsten regression showed no statistically significant trend in mortality (*p* = 0.935). Residual diagnostics confirmed appropriate correction of autocorrelation (Transformed Durbin-Watson ≈ 2), with an initial AR (1) coefficient of −0.52, indicating moderate negative serial correlation prior to model adjustment (Figure 8).

**Figure 9:**
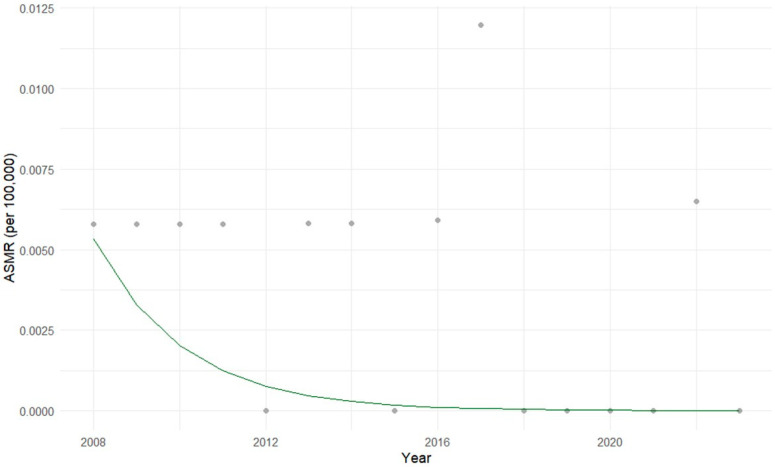
Age-specific mortality rates (ASMR) for hepatoblastoma in adolescents aged 15 to 19 years in Brazil (2008–2023). The gray dots represent the actual ASMR for each year, and the green line represents the mortality trend estimated by the Prais-Winsten regression model. A statistically significant decreasing trend was observed, with an estimated annual reduction of 38.4%.

In adolescents aged 15 to 19 years, the Prais-Winsten regression revealed a statistically significant decreasing trend in mortality (*p* = 0.0159), in contrast to the other age groups. The overall model was significant (*p* = 0.0098) and explained 39% of the variance in the data. Residual analysis indicated no problematic autocorrelation (Transformed Durbin-Watson ≈ 2), with mild negative serial correlation prior to adjustment (ρ = −0.23) (Figure 9).

The APC by age group showed that, among the four age categories evaluated, only adolescents aged 15 to 19 years presented a statistically significant temporal trend. In this group, a mean annual decrease of 67.3% in the mortality rate was observed (95% CI = −85.28–−27.29, *p* < 0.05). In the other age groups (1–4, 5–9, and 10–14 years), the APCs were positive, ranging from 2.6% to 6.1% per year, but with no statistical significance. These findings highlight the statistical instability associated with the rarity of hepatoblastoma, particularly in younger age groups, while also drawing attention to a sharp and meaningful decline in mortality among adolescents.

**Figure 10:**
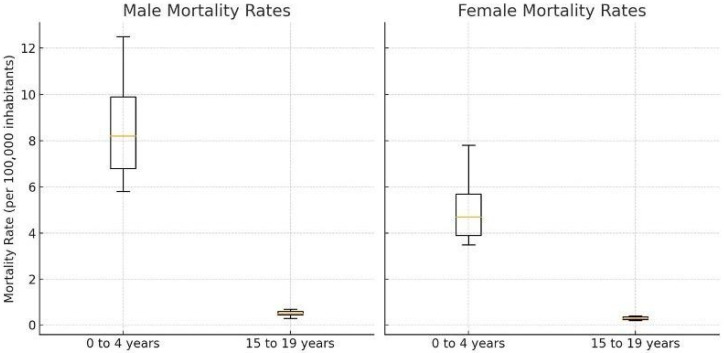
Statistically significant differences in mortality rates were observed between age groups (0–4 years vs. 15–19 years) and between sexes (*p*-values < 0.000001 in both comparisons).

Statistically significant differences in mortality rates were identified between the youngest (0–4 years) and oldest (15–19 years) age groups, as well as between sexes (*p* < 0.000001 for both comparisons). (Figure 10).

### 3.3. Comparative Analysis with Global Data

**Figure 11:**
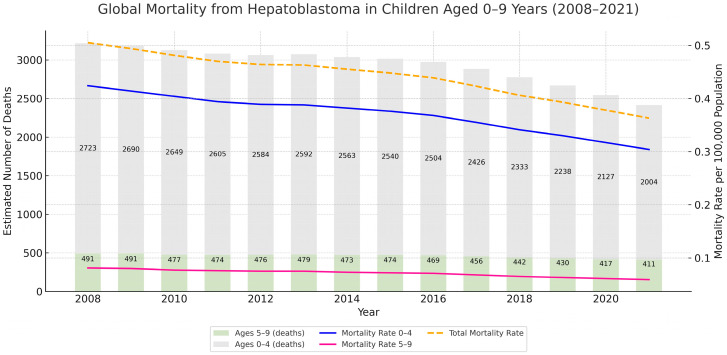
Global mortality by hepatoblastoma in pediatric populations (ages 0–9 years), 2008–2021. Stacked bars represent the estimated number of deaths in children aged 5–9 years (olive green) and 0–4 years (light gray). Overlaid lines indicate ASMR per 100,000 population: 0–4 years (blue), 5–9 years (pink), and total (dashed orange line). Data were obtained from the Global Burden of Disease Study.

Between 2008 and 2021, a global decline in hepatoblastoma-related mortality was observed in children aged 0 to 9 years. As illustrated in Figure 11, most deaths consistently occurred in the 0–4 age years group throughout the study period. Over time, the estimated number of deaths decreased progressively, accompanied by a parallel decline in ASMR across both age groups. The most pronounced reduction was seen among children aged 0–4 year, who initially accounted for the highest burden of disease. Despite overall improvements, mortality rates remained persistently higher in younger children across all years analyzed.

## 4. Discussion

Hepatoblastoma outcomes are inevitably influenced by socioeconomic disparities and the heterogeneous organization of the public health system across regions. Several factors contribute to these differences, including: (1) universal access at the national level, accompanied by regional inequalities; (2) socioeconomic determinants; and (3) the affordability and availability of high-complexity care.

The results of our analysis show that, over the 16-year analysis period, there were 267 reported deaths related to hepatoblastoma in the Brazilian pediatric population. There was a significant predominance among children under 4 years of age, indicating this age group’s heightened vulnerability to the disease. Notably, no statistically significant temporal trend was observed in this age group, suggesting a stable mortality pattern characterized by annual fluctuations around a consistent mean. This may reflect a combination of diagnostic delays, limited therapeutic improvements, and the inherent aggressiveness of hepatoblastoma in early childhood. Mortality rates declined with advancing age, which may reflect not only differences in survival but also the markedly lower incidence of hepatoblastoma among older children. This trend aligns with the recognition that hepatoblastoma accounts for over 90% of hepatic cancers in children aged under 5 years, emphasizing the critical need for early detection and intervention in this younger population.

Studies performed in various countries have revealed similar findings. A systematic analysis by Wu et al., 2021 examined data from the Global Burden of Disease 2021 (GBD), focusing on the incidence and mortality associated with childhood liver cancers from 2001 to 2021 [18]. That study identified hepatoblastoma as the only type of liver cancer affecting children aged 0 to 9 years globally. The analysis highlighted variations in incidence and mortality based on factors such as sex, geographical location, and age groups. Notably, the highest incidence was observed in children aged 2 to 4 years, consistent with the findings of the present study. In contrast, the authors reported an increase in incidence among females aged 12 months to 9 years over the period, within the Brazilian population. Our study revealed a higher incidence of deaths among male patients aged 0 to 19 years, with 164 cases (61.42%), compared to female patients of the same age group, who accounted for 103 cases (38.58%). 

To better contextualize the Brazilian findings within a global perspective, we conducted a direct analysis of global hepatoblastoma mortality data using estimates from the Global Burden of Disease (GBD) Study for the years 2008 to 2021. This timeframe was selected to align with the temporal scope of the Brazilian dataset, allowing a more consistent and meaningful international comparison. The analysis revealed a progressive decline in both the estimated number of deaths and global ASMR throughout the study period, with the most substantial reduction observed among children aged 0 to 4 years, the group that also accounted for the highest absolute burden of the disease. Despite this overall improvement, mortality remained consistently higher among children under 5 years of age, confirming the vulnerability pattern also seen in the Brazilian data. The majority of hepatoblastoma-related deaths in our analysis were recorded among individuals classified as White (45.7%), followed by Brown (43.8%) and Black (5.2%). Smaller proportions were observed in the Indigenous and Ignored categories. While these distributions may partially reflect the demographic profile of the Brazilian pediatric population, they may also indicate potential disparities in access to timely diagnosis, cancer treatment, or the completeness of official mortality records.

Racial disparities in hepatoblastoma incidence and mortality have been highlighted in various studies. Espinoza et al. [19] studied 309 children diagnosed with hepatoblastoma between 1995 and 2018, using data from the Texas Cancer Registry (TCR). Their findings revealed a higher incidence rate among children of Latino origin (5.12%) compared to non-Latino ones (3.15%). Feng et al. [20] on the other hand, analyzed data from the Surveillance, Epidemiology, and End Results (SEER) Program database in the United States. They divided the population into three ethnic groups - Caucasian, African American, and others—and examined 523 hepatoblastoma cases from 2004 to 2015. Their findings indicated a higher incidence and mortality in the African American population, even though the majority of the study ethnic group was of Caucasian origin (76.7%). These findings underscore the need for further research into the genetic, social, and systemic factors that may underlie racial disparities in hepatoblastoma incidence and mortality.

Adjusted models based on our database demonstrated a gradual yet consistent decline in hepatoblastoma mortality across all pediatric age groups. As highlighted before, the highest mortality rates were observed in children aged 0 to 4 years, reflecting the typical early-childhood onset of the disease. Regionally, the Southeast recorded the highest absolute number of deaths, while the Midwest exhibited the most pronounced downward trend over time. These results should be interpreted with caution as they may also result from stochastic variation in low-count time series.

Interestingly, while prior studies have reported poorer survival among adolescents with hepatoblastoma, often attributed to more refractory tumor biology [21,22], our analysis showed a reduction in mortality in this group. This finding should be interpreted with caution, given the small number of cases and limitations of the mortality database. Nonetheless, improvements in diagnostic capacity, treatment protocols, and concentration of care in specialized tertiary centers, particularly in the Southeast and South of Brazil, may have contributed to better outcomes. 

In contrast to the declining trend observed in adolescents, mortality rates among children aged 5–9 and 10–14-years showed no statistically significant temporal variation. The stationary behavior of these time series suggests that year-to-year fluctuations may be the result of the low absolute number of deaths, with limited power to detect subtle temporal changes over time. These findings may reflect that current clinical and public health measures have not substantially influenced mortality patterns in these age groups throughout the study period. Among adolescents aged 15 to 19 years, a statistically significant decreasing trend was identified, with an estimated annual reduction of 38.4%. This distinct decline may reflect improvements in disease recognition, referral systems pathways, or therapeutic strategies that are more effective in older patients. Alternatively, it may indicate greater resilience to the disease or better tolerance to treatment among this age group.

Although this study focused on mortality, it is important to acknowledge that clinical outcomes in hepatoblastoma are heavily influenced by therapeutic strategies, particularly in advanced stages of the disease. In this context, several international multicenter initiatives have contributed to improving disease management over recent decades. For instance, the Société Internationale d’Oncologie Pédiatrique–Epithelial Liver (SIOPEL) group conducted a series of studies emphasizing preoperative chemotherapy and delayed surgical intervention in pediatric patients with hepatoblastoma [23]. SIOPEL-1, a prospective, multicenter international clinical trial conducted between 1990 and 1994, reported a 5-year survival rate of 57% for patients with metastases [24] and an overall survival rate of 75% [20]. This study also introduced the radiology-based PRETEXT I–IV staging system [25].

Other groups such as the Children’s Oncology Group (COG) in the United States, the German GPOH, and the Japanese JPLT adopted the PRETEXT staging system and, in collaboration with SIOPEL, created a unified pediatric hepatoblastoma database. This joint effort led to the establishment of the Children’s Hepatic Tumor International Collaboration (CHIC), producing several important studies, including SIOPEL-6, aimed at mitigating cisplatin-induced hearing loss in treated patients. [25,26]. Single-center studies have also contributed to the characterization of hepatoblastoma in Brazil. Falquetto et al. [27] analyzed 13 cases of primary liver tumors treated at a pediatric hospital between 2012 and 2020 of which 12 of were diagnosed as hepatoblastomas. Most patients were male (66.7%) with a mean age at diagnosis of 2 years, findings that are consistent with the Brazilian epidemiological data presented in this report.

Mortality data were obtained from the Sistema de Informações sobre Mortalidade (SIM), the official national mortality database maintained by the Brazilian Ministry of Health (Ministério da Saúde). This database provides reliable information on temporal and geographical mortality patterns but does not include data on incidence or survival outcomes.

Other limitations of this study stem from its retrospective design and the use of secondary data with limited adjustment for demographic variations, such as population growth and regional disparities. Although adjustments were made to standardize the analysis, such as age stratification, use of mortality rates per 100,000 population and time-series linear regression adjusted using the Prais-Winsten method, residual confounding cannot be entirely ruled out. In addition, the rarity of hepatoblastoma resulted in a small number of events, which may reduce statistical power and increase the influence of outliers. This limitation is particularly relevant in trend analyses, where small fluctuations in case numbers can disproportionately influence the slope of regression models, leading to potential misinterpretation of temporal stability or change. Moreover, data accuracy may be influenced by under-reporting of mortality from hepatoblastoma in Brazil [28,29].

This study evaluated hepatoblastoma-related mortality in the Brazilian pediatric population over a 16-year period, highlighting the highest disease burden among children under five years of age. Although the use of secondary data and the small absolute number of cases, the findings reinforce the crucial role of early diagnosis. When compared with global estimates from the Global Burden of Disease study, this early-age vulnerability appears to be a recurring pattern across different countries, suggesting that the challenges seen in Brazil are not isolated, but part of a broader global scenario. These observations highlight the urgency of strengthening public health responses, encouraging collaborative research efforts, and investing in international strategies that promote more equitable access to pediatric cancer care.

## 5. Conclusions

The global burden of hepatoblastoma has generally declined, but the rate of decline has slowed, particularly in areas with low or middle socio-demographic index [30]. Our study demonstrated that, despite its rarity, hepatoblastoma remains a significant cause of mortality in early childhood in Brazil, particularly among children under five years old, who bear the highest disease burden. The stable mortality rates observed in this age group over the 16-year period suggest limited progress in early diagnosis and therapeutic strategies for the youngest patients. In contrast, the significant decline in mortality among adolescents may reflect improvements in healthcare access, disease recognition, or treatment effectiveness in older age groups. Global data confirmed a similar trend, with declining mortality rates over time, although the youngest children continued to exhibit the highest vulnerability. Racial and regional disparities observed both in Brazil and internationally highlight the urgent need for more equitable public health policies that ensure early diagnosis and high-quality cancer care for all pediatric populations. These findings emphasize the importance of collaborative and global efforts to reduce hepatoblastoma-related mortality, particularly in vulnerable and underserved groups.

## Data Availability

The data that support the findings of this study are available from the corresponding author upon reasonable request.

## Abbreviations

The following abbreviations are used in this manuscript:

APC	Annual Percent Change
AFP	Alpha-Fetoprotein
WNT	Wingless-related Integration Site
CTNNB1	Catenin Beta 1
NFE2L2	Nuclear Factor Erythroid 2-Related Factor 2
TERT	Telomerase Reverse Transcriptase
TP53	Tumor Protein P53
PRETEXT	Pretreatment Extent of Disease
